# Altered Meristem Initiation Is Associated with Increased *OSHB3* Expression in a Semi-Dominant Rice Mutant

**DOI:** 10.3390/biology15110851

**Published:** 2026-05-29

**Authors:** Keisuke Mikami, Momoko Kobukai, Kaito Chiba, Miu Kuwamura, Nobuhiro Nagasawa, Namiko Satoh-Nagasawa

**Affiliations:** Department of Biological Production, Faculty of Bioresource Sciences, Akita Prefectural University, Akita 010-0195, Japann_nagasawa@akita-pu.ac.jp (N.N.)

**Keywords:** meristem, microRNA, rice, *OSHB3*

## Abstract

*HD-ZIP III* genes encode important transcription factors that play a crucial role in plant development and are regulated by microRNAs (miRNA). Although previous studies have examined the functions of rice *HD-ZIP III* genes using overexpression lines, the developmental consequences of increased expression of the rice *OSHB3* gene under native regulatory conditions remain unclear. In this study, we isolated a gain-of-function mutant carrying mutations in the miRNA target site of the *OSHB3* gene, a member of the HD-ZIP III family, and examined its morphology at multiple developmental stages. Gene expression was analyzed using quantitative reverse transcription polymerase chain reaction (qRT-PCR) and in situ hybridization. Our results reveal that ectopic expression of the *OSHB3* gene is associated with the differentiation pattern of meristems in rice and that a correlation exists between *OSHB3* expression levels and phenotypic severity in the mutants. These findings highlight the importance of precise spatial and quantitative regulation of *HD-ZIP III* genes in rice development.

## Simple Summary

The *HD-ZIP III* gene family encodes important transcription factors that play a crucial role in plant development and is regulated by microRNAs (miRNAs). Although previous studies have examined the functions of *HD-ZIP III* genes, the developmental consequences of increased expression of the rice *OSHB3* gene under native regulatory conditions remain unclear. In this study, we analyzed gain-of-function mutants with altered meristem initiation carrying mutations in the miRNA target site of the *OSHB3* gene. In the mutant, we observed elevated and ectopic expression of *OSHB3* and a correlation between expression levels and phenotypic severity. Our findings highlight the importance of precise dosage control of *HD-ZIP III* genes in rice development.

## 1. Introduction

Precise spatial and quantitative regulation of transcription factors is essential for robust plant morphogenesis. Among these regulators, class III homeodomain–leucine zipper (*HD-ZIP III*) genes constitute a family that has undergone evolutionary duplication and increased functional redundancy. These genes act as key determinants of developmental patterning (reviewed in [1]).

Genetic studies have revealed a complex regulatory architecture underlying HD-ZIP III function. In *Arabidopsis* and other species, single loss-of-function mutants often exhibit mild or no visible phenotypes due to functional redundancy among family members, whereas higher-order mutants display severe developmental defects. These findings demonstrate that *HD-ZIP III* genes are required for the establishment of the shoot apical meristem (SAM), maintenance of meristematic activity and organogenesis [2,3,4]. *HD-ZIP III* transcripts are post-transcriptionally regulated by microRNA (miRNA), specifically miR165/166, which restrict their expression domains and help establish developmental boundaries. Mutations in the miRNA target site generate gain-of-function mutants, which produce dramatic and distinct types of abnormalities compared to loss-of-function mutants. Classical mutants such as *phabulosa* and *phavoluta* in *Arabidopsis* and *rolled* in maize carry nucleotide substitutions in the miRNA target sites that impair miRNA-mediated repression and lead to ectopic *HD-ZIP III* expression, resulting in severe leaf polarity defects in those mutants [5,6]. These findings illustrate that although the *HD-ZIP III* gene exhibits a high degree of redundancy in response to decreased expression, even a modest increase in the expression of a single gene can profoundly affect development.

In rice, *HD-ZIP III* genes function has been analyzed using single mutants, RNAi knock-down lines and strong overexpression systems. A single recessive mutant of *OSHB3*, a member of the *HD-ZIP III* family, produced ectopic meristem just after germination [7], whereas an RNAi knock-down of *OsHox33*/*OSHB3* accelerated leaf senescence [8]. Overexpression of miRNA166-resistant versions of the *OSHB3* gene generated ectopic leaf margins, shoots, and radialized leaves, while *OSHB1* overexpression resulted in a milder phenotype with partially adaxialized leaves [9]. A single dominant mutant, *lateral floret 1* (*lf1*) in which *OSHB1* is ectopically expressed, promoted lateral floret meristem development [10]. These results indicate that phenotypic changes resulting from endogenous gene upregulation under normal regulatory conditions differ from those resulting from artificial overexpression. Despite this, the developmental consequences of moderate and spatially altered expression of endogenous *OSHB3* genes under native regulatory contexts remain poorly understood in rice.

Here, we report the isolation of a semi-dominant rice mutant with altered meristem initiation carrying a single-nucleotide substitution in the miRNA target site of *OSHB3*. We observed ectopic expression of *OSHB3* in the mutant, leading to dosage-dependent defects in meristem initiation, with homozygous plants exhibiting more severe phenotypes than heterozygotes. Our findings suggest that fine-tuned dosage control of *HD-ZIP III* genes is critical for maintaining proper meristem patterning in rice.

## 2. Materials and Methods

### 2.1. Plant Materials

The mutant line was isolated by screening M_2_ populations of Taichung 65 (T65) backgrounds treated with N-methyl-N-nitrosourea. Since homozygous mutants are sterile, we used heterozygous plants for maintaining dominant mutations.

For rough mapping, heterozygous mutants were crossed with Kasalath (Ka) cultivars. A total of 44 F_2_ progenies displaying the wild-type phenotype were used for mapping the mutation.

Plants were sown and grown on Murashige–Skoog medium at 28 °C under continuous light conditions after surface-sterilizing and washing with water. Seeds were soaked overnight in a 200-fold diluted solution of fungicide (Kumiai Chemical Industry Co., Ltd., Tokyo, Japan) at room temperature and washed with water. After 2 days in water at 15 °C, the seeds were transferred to a 28 °C incubator overnight and placed in soil after germination.

### 2.2. Genotyping

Genotyping was performed to distinguish homozygous and heterozygous individuals of each mutant. DNA was extracted from individual plants by grinding a small amount of plant tissue in liquid nitrogen with a multi-bead shocker (Yasui Instruments Co., Ltd., Osaka, Japan), adding 300 µL of TPS buffer (0.1 M Tris-HCl pH 8.0, 1 M KCl, 0.01 M EDTA) and incubating at 70 °C for 30 min. For precipitation, samples were centrifuged at 2900 rpm for 15 min at 4 °C. A total of 200 µL of isopropanol was added to the supernatant and the mixture was centrifuged at 4 °C, 2900 rpm for 15 min after mixing. Samples were then washed with 300 µL of 70% ethanol solution followed by a centrifugation at 4 °C, 2900 rpm for 15 min. The pellet was dissolved in 50 µL of sterile water.

PCR was performed using CAPS primers and the PCR products were subjected to restriction enzyme analysis. Primer pairs for each mutant allele and enzymes used for genotyping are listed in Table S1. PCR reactions were performed with KOD neo (TOYOBO Co., Ltd., Osaka, Japan) under the following conditions: initial denaturation: 94 °C for 3 min; (denaturation at 94 °C for 30 s, annealing at 67 °C for 30 s, extension at 68 °C for 90 s) × 6 cycles; (annealing temperature decreased by 0.5 °C per cycle); (denaturation at 94 °C for 30 s, annealing at 62 °C for 30 s, extension at 68 °C for 90 s) × 29 cycles. After the PCR reaction, Mbo I (New England Biolabs, Boston, MA, USA) was used to digest the products and digested samples were electrophoresed to detect polymorphisms.

### 2.3. Mapping

In the first step, we conducted an experiment using pooled DNA and 48 sets of markers to determine the chromosome on which the gene responsible for the mutant was located. Markers located at 12.3 cM, 47.2 cM, 95 cM, and 104 cM on chromosome 12 (which was identified as carrying the causative gene) were used. The regions near 104 cM emerged as candidate regions; therefore, markers were developed at 96 cM and 102 cM to perform a slightly finer mapping. The number of recombinants at each marker position was as follows: 96cM:14; 102cM:1; and 104cM:0. This suggested that the candidate region was likely to be located toward the telomeric end of the long arm of chromosome 12, relative to the marker at 102 cM. The *OSHB3* gene was located within this region.

### 2.4. Sectioning

Samples from the mutant and wild-type plants were fixed with 4% (*w*/*v*) paraformaldehyde (PFA) (Sigma-aldrich, St Louis, MO, USA) and 1% Triton-X (FUJIFILM Co., Tokyo, Japan) in 0.1 M sodium phosphate buffer for 48 h at 4 °C. Samples were then dehydrated in a gradual ethanol series, substituted with t-butanol (FUJIFILM Co., Tokyo, Japan), and embedded in Paraplast Plus (McCormick Scientific, MO, USA). Sectioning was performed at 10 µm thickness using a rotary microtome (Microm, Walldorf, Germany). The sections were stained with hematoxylin (SAKURA Finetek, Tokyo, Japan) and observed under a light microscope (BX-51, Olympus, Tokyo, Japan). For resin sectioning, we followed the same procedure as for preparing paraffin sections up to the 100% ethanol replacement step, then immersed the sample in a mixture of replacement solution (Technovit 7100, Heraeus Kulzer GmbH, Wehrheim, Germany) and ethanol. The concentration of Technovit 7100 was gradually increased every half day until it reached 100%. Subsequently, the resin was poured into a mold and the sample was embedded, covered with parafilm (amcor, Zurich, Switzerland) and placed in the refrigerator overnight to harden. Sections were cut at 5 μm thickness using a microtome and arranged on microscope slides, stained with toluidine blue (Waldeck, GmbH, Munster, Germany), and then observed under a light microscope.

### 2.5. SEM Observation

Samples were fixed in 4% of PFA at 4 °C overnight. Then, they were dehydrated through an ethanol series and isoamyl acetate (KANTO CHEMICAL Co., Ltd., Tokyo, Japan). Subsequently, samples were dried using a JCPD-5 (JEOL Ltd., Tokyo, Japan) critical point drying system. After ion coating, the samples were observed using a TM3030Plus Miniscope (Hitach high-tech, Tokyo, Japan).

### 2.6. RNA Extraction and Quantitative Reverse Transcription Polymerase Chain Reaction (qRT-PCR)

Total RNA was extracted using TRIzol (Invitrogen, Carlsbad, CA, USA), following the manufacturer’s protocol.

A total of 500 µg of RNA was used for first-strand cDNA synthesis using the ReverTra Ace qPCR RT Master Mix with gDNA Remover (TOYOBO Co., Ltd., Osaka, Japan). The cDNA was diluted 20 times and used for real-time PCR. For quantification of the genes, KOD SYBR qPCR Mix (TOYOBO Co., Ltd., Osaka, Japan) was used. The expression level of each sample was normalized to that of an internal control, *UBIQUITIN5* (*UBQ5*). The primers for the detection of *OSHB3/ABL2* and *UBQ5* are listed in Table S1. Amplification conditions were initial denaturation at 98 °C for 2 min, denaturation at 98 °C for 10 s, annealing at 60 °C for 10 s, and extension at 68 °C for 30 s: this cycle was repeated 40 times. Finally, a melting curve analysis was performed. Settings were adjusted to standard settings for each instrument.

### 2.7. In Situ Hybridization

For studying *ABL2/OSHB3* and *OSH1* expression patterns by in situ hybridization, we followed the protocol noted in [9]. Samples were fixed using the same procedure described above.

## 3. Results

### 3.1. Identification of a Semi-Dominant Meristem Mutant Caused by a Single-Nucleotide Substitution in the miRNA Target Site of OSHB3

**Figure 1:**
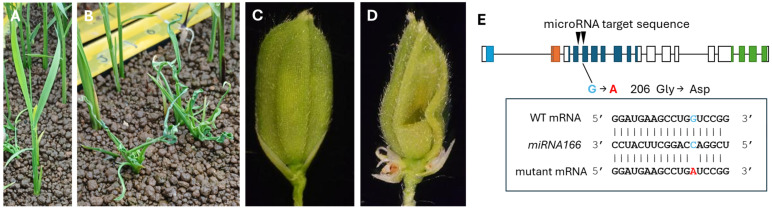
Phenotypic characteristics of *OSHB3* mutants and the gene structure. (**A**) Seedlings of rice wild-type; (**B**) Mutant seedlings; (**C**) Spikelet of wild-type; (**D**) Mutant spikelet; (**E**) Gene structure of *OSHB3* and the mutated site. Light blue box indicates the homeodomain; dark orange box shows the leucine zipper motif; blue boxes contain the START domain; green boxes indicate the MEKHLA domain.

A dwarf, rolled, narrow leaf mutant was isolated, and the mutant plants developed lateral florets during the reproductive stage (Figure 1A–D). In the M_2_ generation, the proportion of abnormal seedlings increased compared to normal seedlings. When the mutants were backcrossed to T65, the segregation ratio of normal to abnormal seedlings in the BC_2_ generation was 33:19, which did not deviate significantly from the expected 3:1 ratio, indicating that it was a dominant mutant. Since *lf1* mutant has been previously reported and the floral phenotypes of our mutant exhibited similar properties, we initially suspected that our mutant is allelic with *lf1* [10,11]. However, sequencing the *LF1* locus did not reveal any mutations in the gene. We then employed rough mapping, which mapped the causative locus of the mutant to the long arm of chromosome 12 where *OSHB3* is located. Subsequently, we identified a mutation that caused an amino acid substitution within the miR166 target sequence at the fifth exon of *OSHB3* that makes up the START domain (Figure 1E).

### 3.2. OSHB3 Transcript Levels Were Elevated in the Mutant

**Figure 2:**
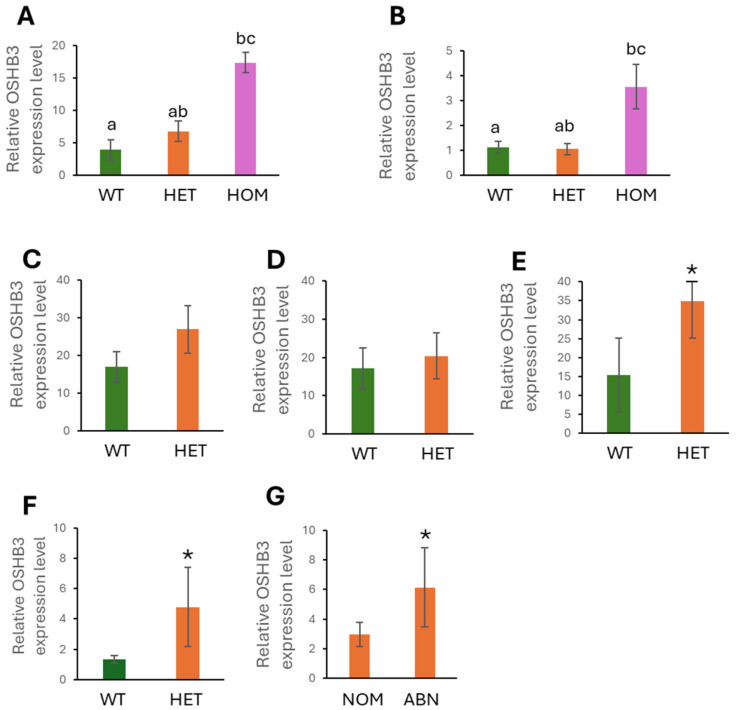
*OSHB3* expression level in wild-type (WT), heterozygous (HET) and homozygous (HOM) mutants. (**A**) Leaves; (**B**) SAMs; (**C**) 1–3 mm panicles; (**D**) 1–1.4 cm panicles; (**E**) 1.5–2.5 cm panicles; (**F**) Flowers; (**G**) Heterozygous normal (NOM) and abnormal (ABN) flowers. Values represent mean ± SD (*n* = 4 in each experiment). Different letters and asterisks indicate significant differences at the 5% level (Tukey–Kramer’s significant difference test and *t*-test, respectively).

To investigate whether a correlation exists between the mutant phenotypes and *OSHB3* expression, we quantified the expression level of *OSHB3* by real-time PCR in leaves, SAMs, and young spikelets. Significant differences in *OSHB3* expression were observed in leaves and SAMs of homozygous plants compared to the control, but the heterozygous plants did not show significant differences in expression (Figure 2A,B). We next analyzed the expression level of *OSHB3* during reproductive development. In young spikelets (1–3 mm and 1–1.4 cm), expression levels in heterozygous plants were comparable to wild-type plants (Figure 2C,D). However, in developing panicles (1.5–2.5 cm), heterozygous plants showed significantly elevated level of *OSHB3* (Figure 2E). The expression level of *OSHB3* in flowers was higher in the heterozygous plants compared to the control (Figure 2F). Among heterozygous flowers, those exhibiting abnormal morphology (e.g., multiple small flowers, absence of lemma or palea) showed higher *OSHB3* expression than heterozygous plants showing nearly wild-type floral architecture (Figure 2G). Taken together, *OSHB3* is strongly overexpressed in the SAM and leaves of homozygous plants and upregulated in the larger developing panicles of heterozygous plants.

### 3.3. OSHB3 Expression Domain Is Expanded in the Mutant

**Figure 3:**
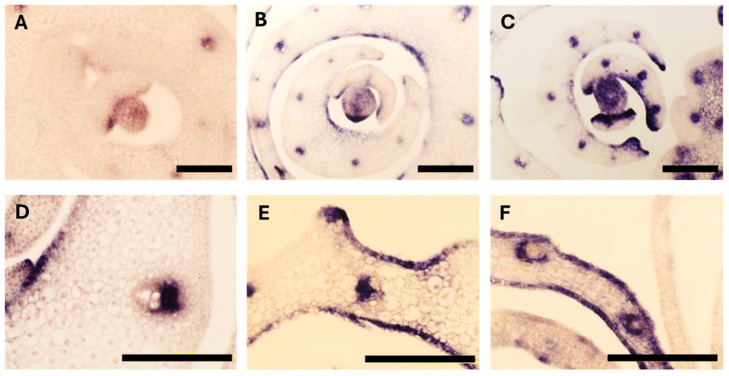
*OSHB3* expression pattern in wild-type, heterozygous and homozygous mutants. (**A**–**C**) Apexes; (**D**–**F**) Leaf sheaths; (**A**,**D**) Wild-type plants; (**B**,**E**) Heterozygous plants; (**C**,**F**) Homozygous plants. Bars: 100 µm. The yellow elements in the image are part of a scale bar automatically added by the digital camera software, so they can be ignored.

We also performed in situ hybridization to examine the expression pattern of *OSHB3* in the mutant. In the wild-type, the *OSHB3* gene was expressed in the meristem, the adaxial surface of young leaf primordium, and the vascular bundle (Figure 3A,D).

In heterozygous and homozygous plants, the signal was stronger and broader at the meristem than in the wild-type (Figure 3B,C). In addition, the signal was observed not only on the adaxial side but also on the abaxial side of developing leaves (Figure 3E,F). The degree of *OSHB3* expression was stronger and broader in homozygous plants than in heterozygous plants and this pattern was also observed around the vascular bundle (Figure 3D–F). During the reproductive stage, ectopic *OSHB3* signals were also observed (Figure S1). Taken together, these results indicate that *OSHB3* is highly expressed in SAMs and ectopically expressed in leaves of homozygous and heterozygous mutants and in spikelet primordia of heterozygous plants.

### 3.4. The Mutant Exhibits Dosage-Dependent Defects in Meristem Initiation

**Figure 4:**
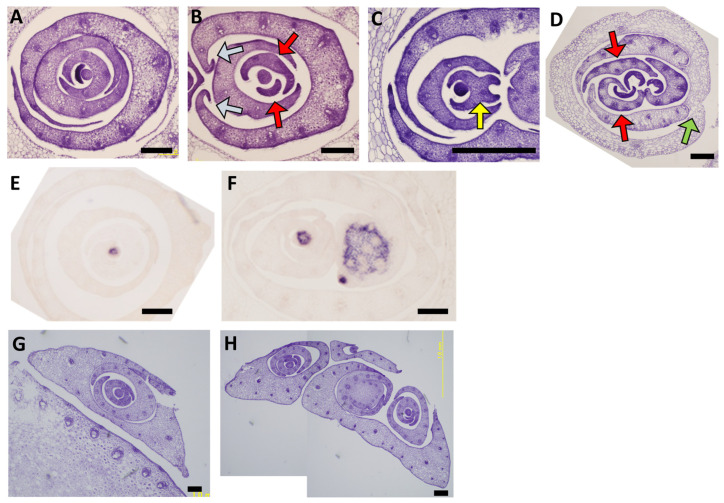
Aberrations in heterozygous and homozygous mutants. (**A**–**D**) Cross sections of vegetative apex. (**E**,**F**) OSH1 expression pattern in vegetative apexes; (**G**,**H**) Structures of tiller buds. (**A**,**E**,**G**) Wild-type plants; (**B**,**C**,**H**) A few representative heterozygous plants; (**D**,**F**) Representative homozygous plants; Bars: 100 µm. The yellow elements in the image are part of a scale bar automatically added by the digital camera software, so they can be ignored.

To investigate the effect of excessive and ectopic expression of *OSHB3*, we examined the mutant phenotype in detail throughout its lifecycle. Cross sections of the apex at 4 days after planting revealed multiple aberrations in heterozygous and homozygous plants, including aberrant phyllotaxy (Figure 4B,D, red arrows), bipolar leaves (Figure 4C, yellow arrow), and leaves in which the sheath leaf and the first leaf were fused together (Figure 4D, green arrow). In some cases leaf margin development was incomplete and leaves were curled inwards (Figure 4B, light blue arrows). As shown in Table 1, the frequency of phenotypic manifestations (in particular, the abnormal leaf arrangement and the formation of ectopic meristems) was higher in homozygous mutants than in heterozygous mutants. Furthermore, the stem arrangement in homozygous mutants was markedly altered (Figure S2), and the plants perished within two months after sowing. Previous studies have shown that aberrant phyllotaxy and bipolar leaves arise from defects in meristem initiation [9]. Therefore, in order to investigate meristem initiation patterns in the mutant, we performed in situ hybridization with *OSH1* as a probe. The analysis revealed the formation of some ectopic new meristems in heterozygous and homozygous plants (Figure 4F, orange arrow head), suggesting defects in the normal meristem initiation pattern.

Next, we examined tiller bud development in heterozygous mutants 3–4 months after sowing. In wild-type plants, tiller buds form a special leaf-like structure called prophyll, followed by new leaves (Figure 4G). Although the mutants also developed prophyll-like structure, they were more complex (Figure 4H). In all of the heterozygous mutants, additional prophylls were observed in the axil of the original prophyll and prophyll formation within a prophyll was also observed. This indicates that ectopic meristems arise not only at the SAM but also within axillary meristems in the mutant.

**Figure 5:**
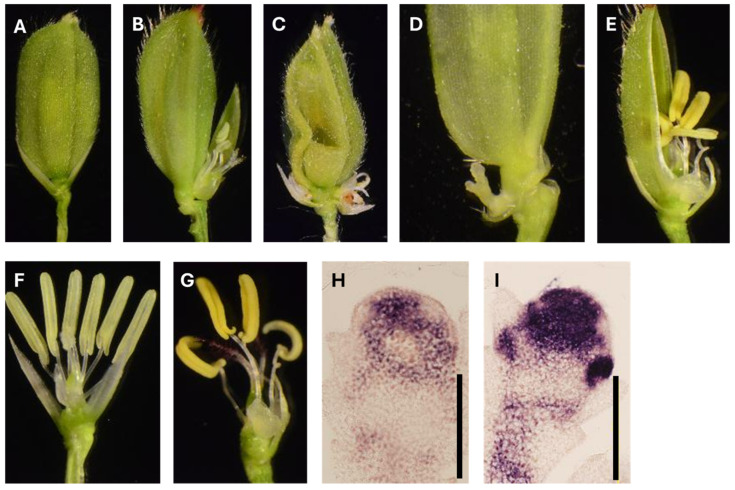
Spikelet structures and floral meristems in representative wild-type and heterozygous mutant plants. (**A**,**F**,**H**) Wild-type plants; (**B–E**,**G**,**I**) Heterozygous plants; (**A**) Normal spikelet; (**B**,**C**) Spikelets with ectopic lateral florets; (**D**) Spikelet with ectopic inflorescence; (**E**) Spikelet without palea; (**F**) Internal structure of a wild-type rice floret showing stamens; (**G**) Internal structure of a heterozygous floret showing reduced number of stamens compared to wild-type (shown in (**F**)). (**H**,**I**) *OSH1* expression pattern in floral meristems of wild-type and heterozygous mutant plants, respectively; Bars: 100 µm. The yellow elements in the image are part of a scale bar automatically added by the digital camera software, so they can be ignored.

Although the frequency of occurrence varied depending on the phenotype, the mutant also exhibited pronounced phenotypes during reproductive growth (Table S2). Specifically, abnormalities such as one or two extra florets, additional inflorescences developed at the axils of sterile glumes, and reduced or extra glumes or palea were observed externally (Figure 5B–E). To investigate the origin of these extra florets and inflorescence, we performed in situ hybridization experiments using *OSH1* to assess meristem initiation. Ectopic *OSH1* signals were detected in the axils of the sterile glumes, providing clear evidence of the newly initiated meristems in the mutant (Figure 5G). In addition, abnormalities in the florets of heterozygous mutants, such as reduced number of stamens and reduced or extra lodicules, were also observed (Figure 5I).

### 3.5. Disruption of Adaxial–Abaxial Polarity in Mutant Leaves

**Figure 6:**
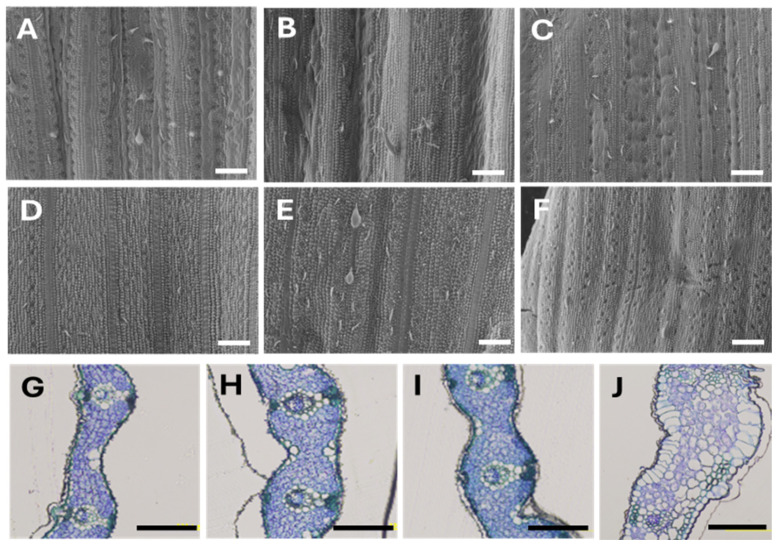
Leaf surface and internal structure of representative wild-type plants and heterozygous and homozygous mutants. (**A**,**D**,**G**) Wild-type plants; (**B**,**E**,**H**,**I**) Heterozygous plants; (**C**,**F**,**J**) Homozygous plants; (**A**–**C**) Adaxial leaf surfaces; (**D**–**F**) Abaxial leaf surfaces; (**G**–**J**) Internal structure of leaf blades. Bars: 100 µm. The yellow elements in the image are part of a scale bar automatically added by the digital camera software, so they can be ignored.

Since previous reports showed that plants ectopically expressing the miR166-resistant version of the *OSHB3* gene exhibit severe defects, including ectopic production of leaf margins, shoots, and radialized leaves, we examined the morphology of leaves in our mutants in detail. Scanning electron microscopy of mature leaves revealed that macro-hairs and micro-hairs, which normally occur on the adaxial surface in wild-type plants, were present on the abaxial side of the leaves in both heterozygous and homozygous mutants (Figure 6A–F). At the boundary between leaf sheath and leaf blade, most heterozygous leaves lacked auricles and some of the homozygous leaves lacked both auricles and ligules. In addition, the boundary between the leaf blade and leaf sheath was slanted rather than horizontal in some homozygous plants (Figure S2). Internal leaf structures, examined by resin sectioning, showed that bulliform cells that are normally restricted to the adaxial side of wild-type leaves were present on the abaxial side and occasionally on both sides in heterozygous leaves (Figure 6H,I). Leaves of homozygous plants were narrower and the arrangement of bulliform cells were highly irregular (Figure 6J). Around the midrib, heterozygous leaves also displayed abnormal vascular bundle alignment (Figure S3). Together, these results indicated that adaxial–abaxial polarity is partially disrupted in the mutants.

## 4. Discussion

The present study suggests that a defining characteristic of a gain-of-function mutant with a single-nucleotide substitution in *OSHB3* is the repeated formation of ectopic meristems adjacent to the primary meristems throughout the plant’s lifecycle.

During early development, both homozygous and heterozygous mutants exhibited phenotypes such as aberrant phyllotaxy and the formation of ectopic meristems, but these phenotypic changes were more frequent in the homozygous mutants (Figure 4A–D; Figure S2; Table 1). Furthermore, homozygous mutants remained dwarf in stature and perished early, whereas heterozygous mutants survived until the reproductive growth stage. The formation patterns and arrangements of axillary meristems in heterozygous mutants were unique. Ectopic meristems differentiated at multiple stages of axillary bud development; in particular, the earliest ectopic meristems formed adjacent to the prophyll at the onset of prophyll formation, and additional ectopic meristems differentiated within the prophylls themselves. As a result, several prophylls developed in parallel or nested configurations (Figure 4H). As they entered the reproductive development, the mutants developed additional phenotypic changes, such as the formation of extra flowers, changes in the number of lemma and palea, and a reduction in the number of stamens (Figure 5; Table S2). In contrast, in the gain-of-function mutant of *OSHB1*, *lf1* [10,11], the only abnormal phenotype observed was the formation of extra flowers, and the lemma, palea, lodicules, and stamens showed normal development. In other words, while both *OSHB1* and *OSHB3* shared a similar function in activating extra meristems in the spikelets during reproductive development, there were differences in the function of these genes during flower development.

The phenotypic changes in the homozygous mutant were more pronounced than those in the heterozygous mutant, and this extent of change correlated with the level of *OSHB3* expression and the size of the expression domain. Among the heterozygous mutants exhibiting individual variation in phenotypic severity, those with visible abnormalities exhibited higher *OSHB3* expression in their flowers than those without (Table S2; Figure 2G). These results suggest *OSHB3* as an important regulator of meristem initiation and underscore the significance of precise dosage control of *HD-ZIP III* genes in rice development. Previous studies using *ACTIN* promoter-driven overexpression of miRNA-resistant *OSHB* genes showed ectopic shoots and aberrant leaf morphology [7], establishing essential roles for *HD-ZIP III* genes in polarity and meristem maintenance during vegetative growth. However, the contribution of *OSHB3* to subsequent developmental processes remained unclear and is addressed in this study.

The contrasting difference in the effects of loss-of-function and gain-of-function mutations in *HD-ZIP III* genes is intriguing. Loss-of-function mutations in individual *HD-ZIP III* genes often produce weak or undetectable phenotypes due to functional redundancy among family members [2,3,4]. In contrast, gain-of-function mutations frequently result in severe developmental abnormalities [5,6]. Both previous studies [9] and our present study show that *OSHB3* mutants follow this pattern. These observations are consistent with the idea that HD-ZIP III activity must be maintained within a relatively narrow range during shoot development. The observation that both the recessive and gain-of-function mutants produce bipolar leaves supports the hypothesis.

Several hypotheses can be proposed to explain the mutation responsible for the phenotype observed in this gain-of-function mutant. First, because the mapping resolution is limited, we cannot exclude the possibility that causal mutations reside outside the *OSHB3* locus and indirectly affect *OSHB3* expression or localization, thereby leading to the observed phenotype. However, the strong correlations between genotypes, phenotypes and extent of gene expression make this unlikely. Second, even if the mutation identified in exon 5 of *OSHB3* is causal, its effect may not necessarily involve miRNA-mediated regulation. The amino acid substitution within the START domain could alter protein function, for example, by affecting dimerization or downstream gene regulation [12], which in turn may influence *OSHB3* expression patterns and contribute to the phenotype. One plausible explanation for the increased and ectopic expression of *OSHB3* involves disruption of miR166-mediated regulation. The mutation we identified is located within the predicted miR166 target site in *OSHB3*. Notably, it corresponds to the sixth nucleotide from the 5′ end of miR166, within the seed region that is critical for target recognition. Mismatches in this region are known to strongly affect miRNA–target interactions [13,14]. Therefore, this mutation may impair miR166 binding, which in turn could cause changes in *OSHB3* expression and contribute to the mutant phenotype. Furthermore, the morphology of the homozygous mutant was very similar to that of *ACTIN* promoter-driven overexpression plants of miRNA-resistant *OSHB3*, in that both were dwarf plants with narrowly rolled leaves [7]. However, ectopic ligules and filamentous leaves that were frequently observed in the overexpression lines were not seen in the homozygous mutants; instead, another phenotype characterized by the absence of leaf auricles was observed (Figure S3). These minor differences could be attributed to the fact that the overexpression lines were generated using the *ACTIN* promoter, but the *OSHB3* expression in the mutants analyzed in this study could be regulated by a miRNA-mediated mechanism.

Taken together, our findings indicate that the dose of *OSHB3* expression can alter meristem initiation patterns in rice. Since the initiation patterns of meristems are related to the overall architecture of plants, the insights we have gained regarding these genes may be useful for understanding how the architecture of rice is regulated throughout its life cycle. From an agronomic perspective, increasing the meristems in the axillary buds can increase the number of panicles, and increasing the meristems in the spikelets can increase the number of grains; therefore, *OSHB3* may represent a potential target for improving rice yields.

## 5. Conclusions

In this study, we identified a semi-dominant rice mutant exhibiting defects in meristem initiation, including ectopic meristem formation, accompanied by elevated and expanded expression of *OSHB3*. The mutant carries a single-nucleotide substitution in the miRNA target site of *OSHB3*. Our findings suggest that precise spatial and quantitative regulation of *OSHB3* is important for proper meristem patterning in rice.

## Figures and Tables

**Table 1:** The frequency of phenotypes in the mutants (%).

Phenotype	Heterozygous Mutants (*n* = 26)	Homozygous Mutants (*n* = 11)
Aberrant phyllotaxy	65	100
Bipolar leaves	27	27
Ectopic meristems	23	100
Fusion of leaves	19	27

## Data Availability

The data presented in this study is available on request from the corresponding author.

## Supplementary Materials

The following supporting information can be downloaded at: https://www.mdpi.com/article/10.3390/biology15110851/s1, Figure S1: *OSHB3* expression pattern in wild type and heterozygous mutants during reproductive growth; Figure S2: Cross section of apex of a homozygous mutant 10-days after sowing. Bar: 1 mm; Figure S3: Leaf blade and sheath boundary phenotype in wild type and heterozygous mutant plants during reproductive growth; Figure S4: Midrib phenotype in wild type and heterozygous mutant plants during reproductive growth; Table S1: Primer information; Table S2: The frequency (%) of abnormal spikelet and flower phenotypes among all flowers in the heterozygous mutants (*n* = 154).

## Abbreviations

The following abbreviations are used in this manuscript:

MicroRNA	miRNA
HD-ZIP III	Homeodomain–leucine zipper III
SAM	Shoot apical meristem
lf1	lateral floret 1
T65	Taichung65
PFA	Paraformaldehyde
qRT-PCR	Quantitative reverse transcription polymerase chain reaction
Ka	Kasalath
*UBQ5*	*UBIQUITIN5*
