# Supplementary material for: Altered Meristem Initiation Is Associated with Increased OSHB3 Expression in a Semi-Dominant Rice Mutant
Source: Biology (Basel). 2026 May 29;15(11):851. doi: 10.3390/biology15110851 (PMC13255657; doi:10.3390/biology15110851)
Supplement: Supplementary file 1 [file biology-15-00851-s001.zip › biology-4289607-supplementary.pdf]

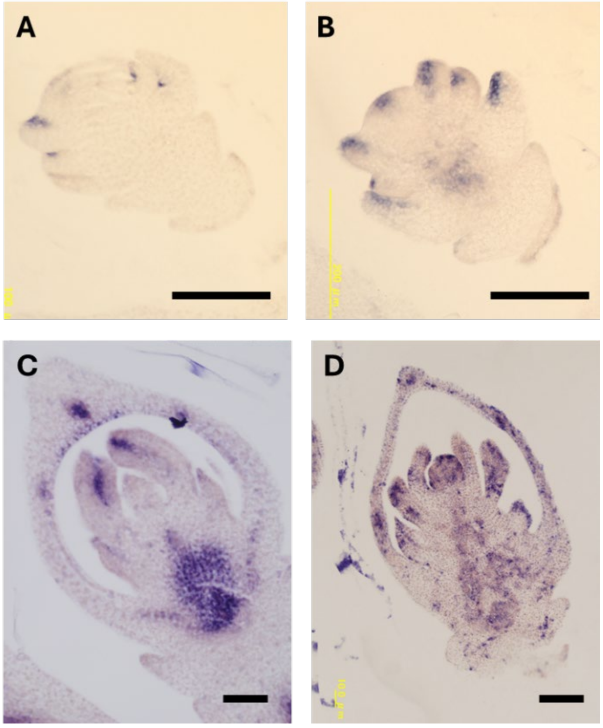


**Figure S1.** *OSHB3* expression pattern in wild type and heterozygous mutants during reproductive growth. (**A,C**) Wild type; (**B,D**) Heterozygous plants. Bars: 100µm.

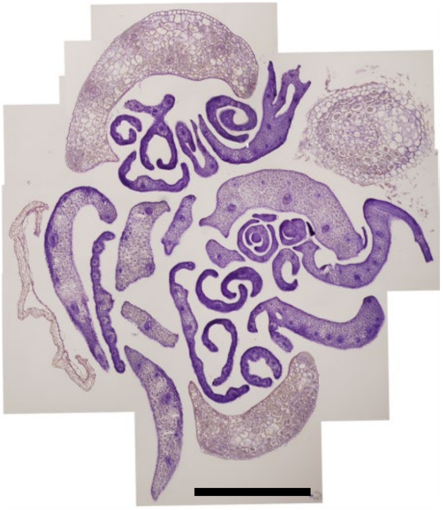

**Figure S2.** Cross section of apex of a homozygous mutant 10-days after sowing. Bar: 1 mm.

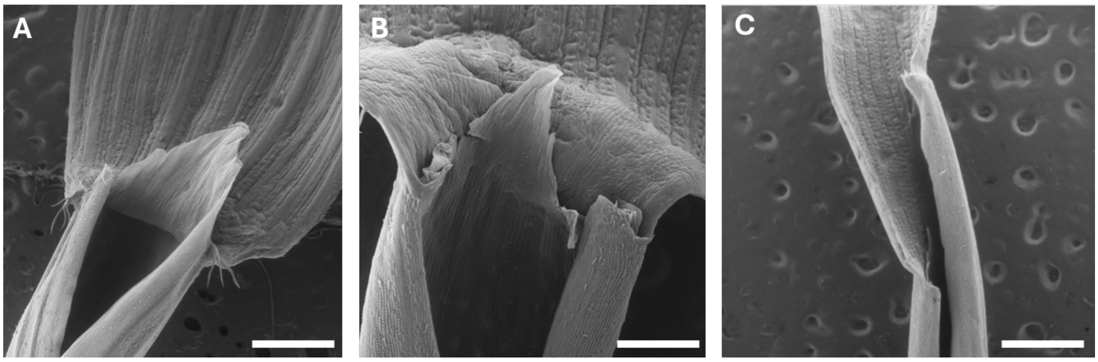


**Figure S3.** Leaf blade and sheath boundary phenotype in wild type and heterozygous mutant plants during reproductive growth. (**A**) Wild type; (**B,C**) Heterozygous plants.  Bars: 1 mm.

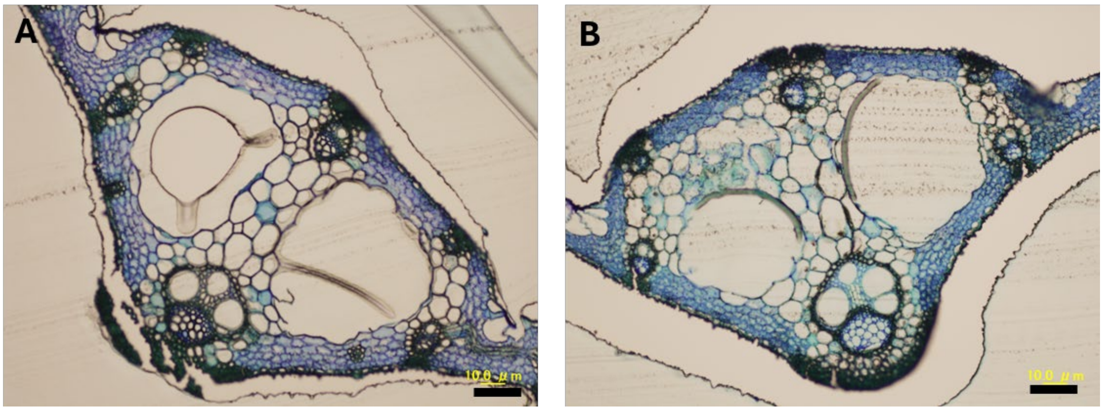


**Figure S4.** Midrib phenotype in wild type and heterozygous mutant plants during reproductive growth. (**A**) Wild type; (**B**) Heterozygous plants.  Bars: 20 μm.

**Table S1**. Primer information.

| | |
|---|---|
| ANS1155 | CCTGTGCTTACAGGCTTTTGTTC |
| ANS1156 | ATGCTTGAGAAAAATGCAACAC |
| ANS1175 | GATACCGAAGATGCCGATTCTC |
| ANS1177 | CTGCATGATCTTGGGGAACTC |
| ANS1080 | ACCACTTCGACCGCCACTACT |
| ANS1081 | ACGCCTAAGCCTGCTGGTT |

**Table S2.** The frequency (%) of abnormal spikelet and flower phenotypes among all flowers in the heterozygous mutants. (n = 154)

| Phenotype | Frequency (%) |
|---|---|
| Reduced empty glumes | 16.2 |
| Extra florets | 26.0 |
| Additional inflorescence | 0.6 |
| Extra lemma or palea | 19.5 |
| Reduced lemma or palea | 8.4 |
| Extra lodicules | 6.5 |
| Reduced lodicules | 36.4 |
| Reduced stamens | 92.2 |
